# Retention, adherence, and acceptability testing of a digital health intervention in a 3-group randomized controlled trial for chronic musculoskeletal pain

**DOI:** 10.1016/j.ctim.2024.103030

**Published:** 2024-03-02

**Authors:** Jennifer Kawi, Chao Hsing Yeh, Lauren Grant, Xinran Huang, Hulin Wu, Chunyan Hua, Paul Christo

**Affiliations:** a University of Nevada, Las Vegas, School of Nursing, 4505 S Maryland Parkway, Las Vegas, NV 89154-3018, USA; b University of Texas Health Science Center at Houston, Cizik School of Nursing, 6901 Bertner Ave, Houston, TX 77030, USA; c University of Nevada, Las Vegas, Kirk Kerkorian School of Medicine, 625 Shadow Ln, Las Vegas, NV 89106, USA; d University of Texas Health Science Center at Houston, School of Public Health, 1200 Pressler Street, Houston, TX 77030, USA; e University of Nevada, Las Vegas, William F. Harrah College of Hospitality, 4505 S Maryland Pkwy, Las Vegas, NV 89154, USA; f Johns Hopkins University, School of Medicine, 733 N Broadway, Baltimore, MD 21205, USA

**Keywords:** Chronic pain, Acupressure, Exchange, Health Information, Application, Mobile

## Abstract

**Objectives:**

Evaluate a digital health intervention using Auricular Point Acupressure (APA) for chronic musculoskeletal pain in terms of participant retention, adherence, acceptability, and satisfaction. Chronic musculoskeletal pain is a global concern and there are persistent challenges in pain management. Despite the value of digital health interventions, these interventions need to be fully evaluated for feasibility.

**Methods:**

We conducted a 3-group, longitudinal, randomized controlled trial (RCT). After Institutional Review Board approval, we posted recruitment flyers in a university, healthcare clinics, and community settings. Participants were randomized into an in-person + app group (*n* = 8), virtual + app group (*n* = 7), and a wait-list, education-enhanced control group (*n* = 8), evaluating our outcomes using standard feasibility measures. The 4-week intervention consisted of virtual sessions, telecommunications, and our APA app, followed by a 3-month follow-up.

**Results:**

Data from 22 participants were subsequently analyzed (95.7%). All app participants adhered to the study protocol and used APA at the minimum recommended frequency and duration. The virtual + app group used APA more during the intervention and follow-up periods. All app participants found the intervention to be acceptable and at least 80% overall were satisfied with APA at the 3-month follow-up. There were no adverse events reported.

**Conclusions:**

Our digital health intervention was found to be acceptable and sustainable; participants adhered to and were satisfied with the intervention providing support for a larger RCT.

**Clinical Trial:**

#: NCT05020470

## 1. Introduction

Musculoskeletal pain is a persistent, global concern affecting approximately one third to almost half of individuals worldwide, resulting to one of the leadings causes of disability.^1–3^ Musculoskeletal pain refers to discomfort in the muscles, bones, joints, and contiguous connective tissues.^1^ Despite multiple nonpharmacologic pain interventions and advances in pharmacologic management, chronic musculoskeletal pain remains substantially prevalent resulting to enormously high indirect and direct healthcare costs. Chronicity in pain denotes a timeframe where the discomfort persists for at least three months or most of the days in the past six months.^4^

The current global economic burden and healthcare costs associated with chronic musculoskeletal pain points to the necessity to test interventions that can be feasible and then evaluate these for efficacy. Digital health interventions have the potential to improve pain and pain care delivery while being largely scalable. Digital health includes various approaches involving technology such as applications (app), wearable devices, mobile health, telehealth, health information technology, and personalized medicine ^5^ Leveraging digital health, we conducted a study to evaluate retention, adherence, acceptability, and satisfaction of our non-pharmacological intervention, Auricular Point Acupressure.

## 2. Background

**Fig. 1.**
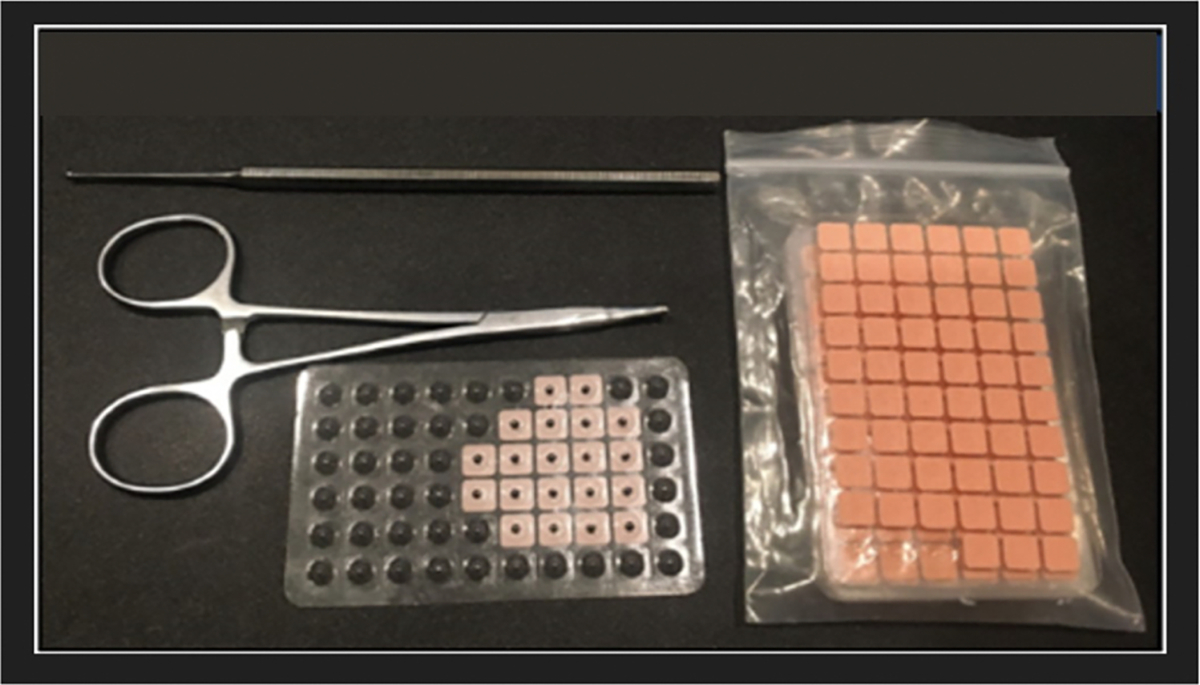
APA Kit. *Note.* Fig. 1 is our APA kit. From “Evaluating Auricular Point Acupressure for Chronic Low Back Pain Self-Management Using Technology: A Feasibility Study,” by C. H. Yeh, J. Kawi, A. Ni, and P. Christo, 2022, *Pain Manag Nurse, 23*(3), p. 303. Fig. 2 is a sample image from the App for low back pain.

**Fig. 2.**
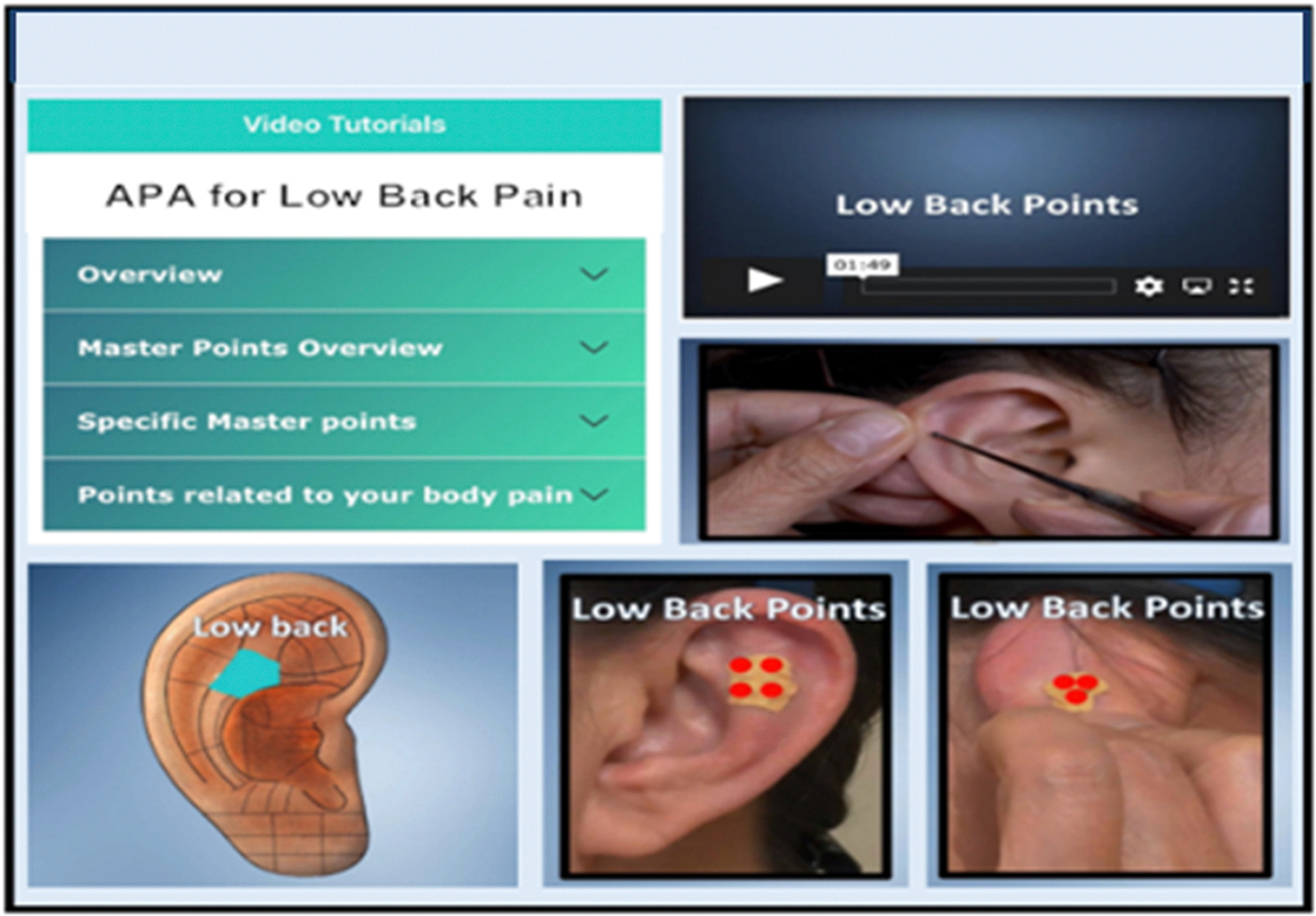
App for Chronic Low Back Pain.

Auricular Point Acupressure (APA) is grounded on the science supporting acupuncture which is an evidence-based modality for chronic pain, especially for low back pain.^6^ However, acupuncture is limited in its scalability and access to the population because of inadequate number of trained providers, several visits required for treatment, and the need to use a “needle,” albeit tiny in gauge. APA uses similar stimulation points as in acupuncture but instead of needles, APA utilizes small pellets (0.2 ×0.2 mm size, Fig. 1) or Vaccaria seeds that are taped securely into ear points (pressure points on the ear) based on body areas of pain. For example, the ear points for low back pain is in the upper region of the antihelix body below the superior crus, a ridge in the upper part of the ear (Fig. 2). Patients use a probe to stimulate the low back zone in the ear to locate the tender ear points and then apply the pellets which are pressed at a standardized minimum dosage of least three times a day for three minutes each time (total of at least 9 min per day) to decrease pain level.^7^ When patients feel mild discomfort or localized tingling on their ear point, then optimal pressure is achieved.^7,8^

APA has evolved into modern science, derived from traditional Chinese medicine, based on the works of Paul Nogier, MD since the 1980 s^9–11^ With multiple experimentation, he developed a somatic map of the body so that certain points in the ear correspond to areas of the body. Hence, there are ear points specific to each pain location or disease. These ear points are confirmed by the presence of tenderness when stimulated either through needles as in auricular acupuncture, electrically, or using pellets as in APA. Correlations between ear points and brain pathways have been validated by functional magnetic resonance imaging.^12,13^ It is theorized that there are nerves in the outer ear corresponding to certain areas of the brain which have a complex connection with specific body parts.^14,15^ Stimulation of these ear points result to the brain being induced to correct its pathological reflex centers,^12,13,16^ changes in serum inflammatory cytokine levels by decreasing pro-inflammatory cytokines and increasing anti-inflammatory cytokines,^17–20^ and reflex reactions in the body to relieve pain.^7,14,15^

Our interdisciplinary team has accumulated extensive evidence in testing APA in clinical trials, compared to sham APA, and demonstrated the following: (1) APA resulted to ≥ 38% rapid pain relief three minutes post-APA^21,22^ and > 44% sustained pain relief as well as > 28% improved physical function at follow-up after 4 weeks of APA;^23–29^ and (2) There were ≥ 60% of participants who reported less use of pain medications after 4 weeks of APA.^24,30^ Due to the need to facilitate wider implementation of APA, we developed a self-guided, mobile-enabled APA app which allowed participants to self-administer APA. Testing this app after 4 weeks, participants reported decreased pain intensity (30%), reduced pain interference (35%), decreased disability (40%), and better physical function (47%).^31^

With the coronavirus-19 pandemic that started in 2020, we pivoted our ongoing chronic pain study using APA from in-person visits to secured virtual connections. We developed a protocol to conduct a telehealth-similar approach to providing APA instructions and have participants self-administer APA in a multisite study with the assistance of the APA app. We found that APA self-administration with the app was doable, acceptable, and beneficial to the participants^32^ and resulted to decreased pain intensity (29%) after a 4-week intervention^33^.

Using feedback from providers and participants,^31–33^ we advanced the APA app through a user-centered design, an iterative process addressing participants’ needs. Our research team made the following improvements: (1) Shortened the video tutorials to make them brief and more easily accessible; (2) Created step-by-step content detailing how to locate ear points and how to stimulate these using graphic videos and images; (3) Expanded content using brief 1–3 min videos, PowerPoints, and images showing ear points on various musculoskeletal areas of pain; (4) Added easily readable instructions sheets; and (5) Created a self-monitoring dashboard that portrays visual graphics of pain statistics and APA usage based on participants’ daily progress and responses to their ecological momentary (real time) assessments of their pain and pain-related outcomes.

Based on our previous studies,^31–33^ we next needed to test this promising, and newly-refined app in a randomized controlled trial (RCT) using 3 groups: in-person + app group, virtual + app group, and a wait-list, education-enhanced control group. Evaluating an in-person compared to a virtual group is necessary to see if APA can be further scaled up to reach a wider population without needing in-person visits. In addition, having a wait-list control group allowed participants the opportunity to go through the intervention after being wait-listed. Therefore, consistent with critical recommendations in the process of intervention testing,^34,35^ our research objectives were to evaluate participant retention, adherence, acceptability, and satisfaction of our digital health intervention which consisted of the app, virtual sessions, and telecommunications.

## 3. Methods

### 3.1. Design

This study was a prospective, longitudinal, 3-group RCT. Institutional Review Board approval (IRB00290512) was received. This manuscript was written following the Consolidated Standards of Reporting Trials, focused on the research objectives aforementioned; other data will be published elsewhere due to the quantity of data gathered.

### 3.2. Participants, Study Setting, and Procedures

This study was conducted at a university, healthcare clinics, and community settings in the west coast. A recruitment flyer was used to advertise the study in November 2021. The following were the inclusion criteria: (1) 18 years or older, (2) able to read and write English, (3) had chronic musculoskeletal pain persisting for at least 3 months or most of the days in the past 6 months, (4) reported an average pain intensity of ≥ 4 on an 11-point numerical pain scale for the previous week, (5) had a smartphone, and (6) able to apply pressure to the seeds taped to their ears. Based on the global description of musculoskeletal pain,^1^ participants with pain in the following areas or pain locations were included in the study: back, neck, shoulder, elbow, wrist, hand, hip, knee, ankle, and foot. A pain intensity of 4 was used because it is the minimum required in most clinical trials and lower numbers are found to be associated with less interference in function.^36,37^ Participants were excluded if they had any allergy to latex due to the tapes used to secure the pellets on their ear points.

**Fig. 3.**
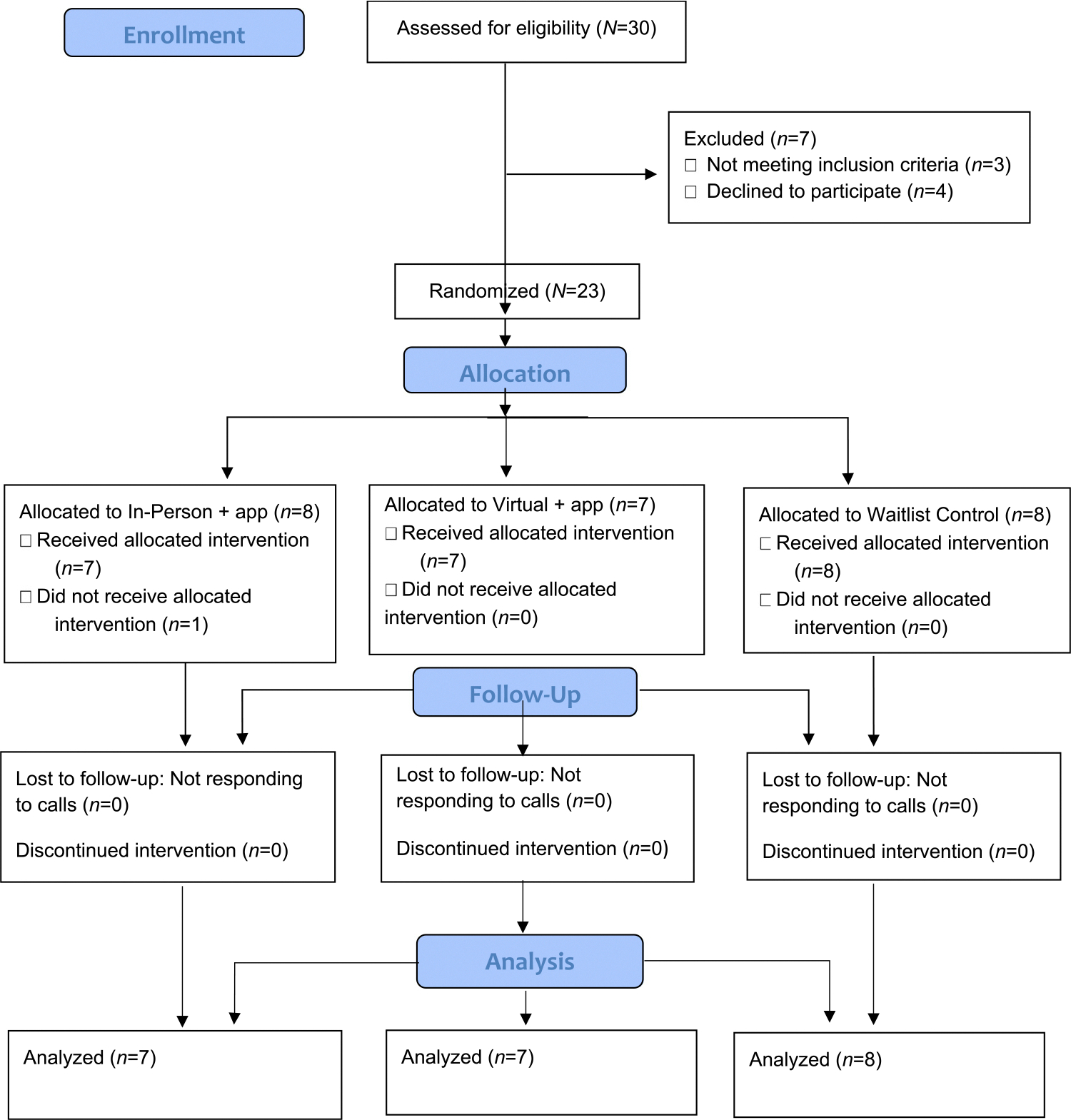
Flow Diagram.

Immediately after study advertisement in November 2021, potential participants contacted the trained study coordinator to express their interest in the study; recruitment continued for two months thereafter to a total of 30 potential participants assessed for eligibility (See Fig. 3). This is a common sample size for pilot studies needed to address study objectives reasonably,^34,35^ as we have also demonstrated in our prior studies.^31–33^ Seven participants were excluded because they either did not meet the inclusion criteria (*n* = 3) or declined to participate after they expressed their initial interest (*n* = 4). Informed written consents were obtained. Using a computer-generated randomization tool, the study coordinator randomized 23 participants into 3 groups in blocks of 3 and 6 with 1:1:1 allocation: in-person + app group (*n* = 8), virtual + app group (*n* = 7), and wait-list control group (*n* = 8). The principal investigators were blinded to the group assignment.

The 2 app groups underwent a 4-week intervention and then a 3-month follow-up. The in-person + app group received in-person instructions and training on APA and the app at the recruitment site for approximately 15–20 min while the virtual + app group received similar instructions and training via secured virtual connection for a similar amount of time. Depending on the location(s) of pain for each app group participant, they were shown the specific video in the app demonstrating ear points on their pain location(s). For example, a participant with neck and low back pain is shown the specific videos in the app that demonstrate ear points for the neck and low back.

After 3 days, all app group participants emailed their ear photos showing the placements of pellets into their ear points specific to their areas of body pain. These ear points were verified for accuracy by an acupuncturist and corrections were provided to each participant. A second set of photos were requested to ensure accuracy of placements when needed. Within the first week of the intervention, a virtual call for approximately 15 min was also conducted with each app participant by the acupuncturist. This allowed for further verification of accuracy of placement of pellets into specific ear points for pain relief and provision of self-management support and answers to APA questions. Participants continued with APA use at the minimum recommended dosage of at least three times per day for three minutes each time (or more when needed for pain) during the 4-week intervention for 4 weekly cycles. Each cycle consisted of 5 days of APA use and 2 days off to allow the ears to desensitize from the pellets. Structured weekly telecommunications were done during the intervention period using APA protocol reminders with the opportunity for questions and answers. Those with 3 or more areas of pain locations had their choice of top 3 pain areas treated. Participants were then followed for 3 months after the 4-week intervention. During the follow-up periods, participants were advised to continue using APA as needed for their pain. Structured monthly telecommunications continued during the 3-month follow-up periods to facilitate retention and provide further opportunities for questions and answers. The app groups did not receive any pain self-management education.

The control group received 4 weeks of pain self-management education which we have effectively used in the past to control for time and attention.^38,39^ Specific information included topics such as types of pain, different treatment modalities, making treatment plans, managing medications, communicating with providers, and self-management strategies. The control group was re-randomized into either of the app groups at the end of their 2nd month of participation in the study, received their assigned intervention on their third month for 4 weeks, and then underwent a 3-month follow-up, with procedures similar to the app groups. All participants continued with their usual care.

### 3.3. Measures

#### 3.3.1. Demographics and other relevant variables

A self-report survey was used for age, gender, race, ethnicity, employment, and education level. Other relevant data were also collected such as smoking, BMI, duration of musculoskeletal pain, pain sites, and pain intensity measured using the standard numerical rating scale of 0 (no pain) to 10 (pain as bad as you can imagine).^40^

#### 3.3.2. Retention, adherence, acceptability, and satisfaction using standard feasibility measures.^34,35^

##### 3.3.2.1. Retention.

This refers to treatment-specific retention rate for each group at baseline and all time points.

##### 3.3.2.2. Adherence to Study Protocol.

Adherence is defined as pressing the pellets for at least three times per day (frequency) at three minutes each time (duration), totaling at least 9 min per day. This also includes the proportion of participants who continued to use APA (yes or no) on their own after the four-week intervention period and monthly follow-ups. Lastly, we evaluated the proportion of participants who completed the measures at each time point.

##### 3.3.2.3. Acceptability.

This was measured using a self-report item asking the participants as to the difficulty level of practicing APA from 1 (no difficulty) to 5 (extremely difficult) in terms of frequency, duration, and time of day. A lower number indicates treatment acceptability.

##### 3.3.2.4. Satisfaction.

This was measured using a self-report item asking participants whether they were satisfied with the intervention. Participants marked their best choice from not satisfied, somewhat satisfied, or completely satisfied. Reflective of their satisfaction, participants were also asked whether they would recommend APA to their family (yes/no).

### 3.4. Data Collection

We collected data from each group at pre-intervention (baseline, first time point), and at 1-month (second time point), 2-month (third time point), and 3-month follow-ups (fourth time point). With instructions at the first time point, app group participants installed their APA app onto their smartphone. They also received instructions on how to use the app to administer APA for 4 weeks and proceeded with the intervention.

### 3.5. Data Analyses

Descriptive statistics were used to analyze the data including means, standard deviation, range, frequencies, and percentages. To prevent any bias due to the control group being wait-listed, we did not include the control group in the analyses once they were re-randomized. Their data may reflect inflation of the outcomes from the intervention as participants may wait to address their pain until they are re-randomized. However, we report the number of participants in the control group who were re-randomized into either intervention group and subsequently completed the study.

## 4. Results

The mean overall age of our participants was 43, 78% were female, 57% were White, and majority (78%) were not Hispanic (Table 1). Almost half were unemployed (43%) but many were college graduates (61%). There were 39% who were either current or previous smokers with an overall BMI of 24.93. The duration of chronic musculoskeletal pain was 1–5 years (48%) or more than 5 years (48%); majority (73%) had 3 or more areas of musculoskeletal pain. The mean pain intensity at the primary location of their pain was 5.9 out of 10 and the back (57%) was the most common primary pain location.

In a short period of two months, we were able to recruit 23 participants (out of 30 assessed for eligibility). We successfully randomized all participants into 3 groups without challenges.

### 4.1. Retention, adherence, acceptability, and satisfaction

During the intervention period, we were able to retain all but one participant who changed his mind immediately after baseline data were collected accordingly due to his busy schedule (95.7% initial retention; Table 2). This participant was in the in-person + app group. We also lost another participant in the in-person + app group at the 3-month follow-up (did not respond to communications; 87% final retention). All other participants were retained in the study. Further, the control group participants who were re-randomized into either app groups were fully retained until study completion up to the 3-month follow-up.

For adherence to study protocol among the app group participants during the intervention period, all used APA at the minimum recommended frequency and duration of at least 3 times for 3 min each time to a total of at least 9 min per day (Table 3). However, the virtual + app group used APA more at 4 times, 3.7 min each time, to a total of 13.6 min per day during the 4-week intervention period. Overall, after the 3 months follow- up, the virtual + app group used APA more frequently at a mean of 3.6 times for 3.2 min each time and total of 11.7 min per day compared to a mean of 2.5 times for 2.7 min each time to a total of 7.6 min per day in the in-person + app group.

As for the proportion of participants who continued to use APA among the app groups after the 4-week intervention, overall, 73% participants continued to use APA from immediate post intervention period to the 3-month follow-up (Table 4). More participants (86%) in the virtual + app group continued to use APA at the 3-month follow-up compared to 64% in the in-person + app group with some missing responses. In terms of overall completion of study measures, these were fully completed at 100% at baseline, 91% at 1-month follow-up, and 87% at 3-month follow-up (Table 5).

In the area of treatment acceptability (assessed in terms of difficulty level from 1 [not difficult] to 5 [extremely difficult]) at the 3-month follow-up, the in-person + app participants rated this at 1.8 for frequency and duration using APA (Table 6). The virtual + app participants rated their difficulty level at the 3-month follow-up at 1.9 in terms of frequency using APA and 1.4 for duration using APA.

For overall APA satisfaction, 87% participants felt somewhat to completely satisfied at the 1-month follow-up to 80% at the 3-month follow-up plus missing responses (Table 7). No participant was unsatisfied in either app group at the 3-month follow-up. Consistent with this, 80% of the participants would like to recommend APA to their family at the 3-month follow-up (plus 20% missing responses; Table 8). Further, no adverse effects were reported by any of the participants.

## 5. Discussion

Digital health interventions, particularly apps, are becoming increasingly common. Especially during the height of the pandemic, digital health solutions have helped to minimize health care interruptions.^41^ In our protocol, we maximized the use of telehealth sessions, telecommunications, and our user-centered app, developed and redesigned with significant participant feedback.^31–33^ However, it is important to evaluate the feasibility of digital health interventions especially in pain studies.

At least 43% of our participants were non-white or mixed race and unemployed. This is important because non-whites and those who are socioeconomically disadvantaged are commonly underrepresented in pain studies resulting to pain care disparities especially when they are documented in the literature to face more severe pain and pain-related disabilities, and are less likely to receive adequate pain treatment.^42–45^ A significant majority of our participants (73%) also had 3 or more areas of chronic musculoskeletal pain with moderate pain intensity. It is very important to be able to reach participants with multisite pain in chronic pain studies because multisite pain is increasingly more prevalent than single pain location.^46^

Several studies and systematic reviews have evaluated pain apps with a variety of use (e.g., pain diary, assessment, education, self-management, cognitive behavior therapy, exercise)^47–50^ and in general, many have been found beneficial especially in the outpatient setting.^51^ However, there are existing problems as to usability, clinician engagement in app development, end-user involvement, and lack of rigorous testing.^47,49,50,52,53^ Our study aimed to address these concerns. Healthcare providers (e.g., acupuncturist, nurse practitioner, and physicians) as well as participants were heavily involved in our app development and re-design. The app and our study protocol has also undergone various randomized controlled trials^31–33^ apart from this current study results.

Critical information has been lacking on logistical feasibility, recruitment, and retention in scientific literature for complementary interventions.^54^ In this study, we were able to successfully recruit participants in a short period of time even during a holiday period (Thanksgiving and Christmas time), randomize them into three groups, and retain them, including our participants in the control group using education-enhanced strategies and wait-listing. Moreover, we were also able to recruit disparate populations while being able to address multisite pain which are common challenges in many pain studies. No attrition was found among the participants in the virtual + app and control groups with a total of 2 participants lost in the in-person + app group.

Intervention refinement and optimization (e.g., appropriate dosing, frequency, duration, intervention delivery) have also been reinforced to facilitate rigorous clinical research in complementary interventions.^54^ Through our multiple, independent studies, we fully developed and comprehensively refined our intervention in an iterative process, and standardized and manualized our intervention protocol to allow for replication fidelity.

Further, feasibility outcomes^34,35,54^ are necessary to be able to move forward to a large scale, efficacy trial and implementation studies. Our app group participants all adhered to the study protocol and minimum recommended frequency and duration of APA use although the virtual + app group had a higher frequency and duration of APA use. The virtual + app group also continued to use APA more than the in-person + app group during the 1 to 3 months of follow-up. These data indicated that the study intervention could be well-delivered virtually.

Participants in both the in-person and virtual + app groups found APA to be acceptable or very minimally difficult with 87% overall who were satisfied. These were sustained until the 3-month follow-up and importantly, no one was found to be dissatisfied with a great majority interested in recommending APA to their family.

Overall, compared to the in-person + app group, participants in the virtual + app group had higher retention, APA adherence, satisfaction, continued use after the 1-month intervention, and greater likelihood recommending APA to their families, all sustained at the 3-month follow-up. However, they found APA to be more difficult initially which improved at the 3-month follow-up. It is possible that because participants had to learn APA remotely, this was more difficult initially compared to those who had an in-person visit. As they repeated the intervention in time, participants reported ease of use similar to those in the in-person + app group. Our findings demonstrate that APA is feasible remotely using technology and showed even better responses for the virtual + app group likely due to convenience and easier access, without in-person requirement.

Other pain-related apps continue to be lacking in quality, rigorous testing, healthcare provider feedback, or end-user involvement in development and refinement.^47–53^ Our digital health intervention overcame these challenges. All our findings reinforce readiness for APA using our study protocol to be implemented in large RCT and pragmatic clinical trials.

In summary, chronic musculoskeletal pain continues to be a highly prevalent condition with persistent difficulties in effective pain management. APA, derived from the principles of acupuncture which is an evidence-based modality for pain, is more accessible to patients and cost-effective as it can be self-administered. To increase scalability of APA, we leveraged technology. Digital health interventions are commonly used in practice but there remain continued challenges in the use of apps. Our interdisciplinary team developed, refined, and rigorously tested our user-centered APA app based on patient and provider feedback resulting in successful feasibility outcomes.

## 6. Limitations

Due to the nature of our study, participants were not blinded so potential bias can be a concern. It also lacks generalizability with a small sample size although small sample sizes are common in pilot studies.^34^ Further, there were 7% (1-month follow-up) to 20% (3-month follow-up) missing responses particularly with respect to APA satisfaction and recommendation to family; we cannot assume how these participants would have responded.

## 7. Conclusions

Using a RCT, we were able to effectively recruit and randomize our participants in a short period of time with high retention. All participants adhered to the protocol, found our intervention to be acceptable, and were satisfied overall with APA without adverse events reported. Moreover, we were able to effectively address multisite chronic musculoskeletal pain instead of a single area of pain location where other pain modalities may be limited.

## Figures and Tables

**Table 1:** Characteristics of the participants (N = 23).

Baseline characteristics	In-person + app	Virtual + app	Waitlist Control	Overall
				
	n = 8	n = 7	n = 8	n = 23

Age, years				
Mean (SD) (Range)	36.4 (11.80) (21–53)	44.9 (19.95) (19–75)	48.3 (16.13) (20–70)	43.1 (16.21) (19–75)
Gender (n, %)				
Female	7 (88)	5 (71)	6 (75)	18 (78)
Male	1 (13)	2 (29)	2 (25)	5 (22)
Race (n, %)				
Asian	1 (13)	1 (14)	1 (13)	3 (13)
Black or African-American	1 (13)	2 (29)	0 (0)	3 (13)
More than one race	1 (13)	1 (14)	2 (25)	4 (17)
White	5 (63)	3 (43)	5 (63)	13 (57)
Ethnicity (n, %)				
Hispanic or Latino	2 (25)	1 (14)	1 (13)	4 (17)
Not Hispanic or Latino	6 (75)	6 (86)	6 (75)	18 (78)
Prefer not to say	0 (0)	0 (0)	1 (13)	1 (4)
Employment Situation (n, %)				
Working (full time)	8 (100)	1 (14)	4 (50)	13 (57)
Not employed	0 (0)	6 (86)	4 (50)	8 (43)
Education Level (n, %)				
High School	0 (0)	0 (0)	0 (0)	0 (0)
Some college	1 (13)	3 (43)	1 (13)	5 (22)
College Graduate	4 (50)	4 (57)	6 (74)	14 (61)
Post-graduate degree	3 (37)	0 (0)	1 (13)	4 (17)
Smoking (n, %)				
Current smoker	0 (0)	1 (14)	2 (25)	3 (13)
Never smoked	7 (88)	4 (57)	3 (38)	14 (61)
Previously smoked, but quit	1 (13)	2 (29)	3 (38)	6 (26)
BMI Mean (SD)	26.57 (8.14)	19.76 (10.23)	27.81 (5.86)	24.93 (8.54)
Duration of musculoskeletal pain (n, %)				
6 months to 1 year	0 (0)	1 (14)	0 (0)	1 (4)
1–5 years	5 (63)	2 (29)	4 (50)	11 (48)
More than 5 years	3 (38)	4 (57)	4 (50)	11 (48)
Number of pain areas or locations related to musculoskeletal pain (n, %)				
1	2 (25)	0 (0)	0 (0)	2 (9)
2	2 (25)	2 (29)	0 (0)	4 (17)
3	2 (25)	2 (29)	6 (75)	10 (43)
4	0 (0)	1 (14)	1 (13)	2 (9)
5	0 (0)	1 (14)	0 (0)	1 (4)
more than 5	2 (25)	1 (14)	1 (13)	4 (17)
Pain intensity (Back): Mean (SD)	5.6 (1.69)	5.1 (2.04)	6.9 (1.25)	5.9 (1.76)
Pain intensity (Neck): Mean (SD)	5.0 (1.26)	5.0 (1.26)	6.5 (1.60)	5.6 (1.54)
Pain intensity (Foot): Mean (SD)	4.5 (0.58)	5.0 (1.73)	5.0 (2.45)	4.9 (1.88)
Primary pain location (n, %)				
Back	4 (50)	4 (57)	5 (63)	13 (57)
Neck	1 (13)	1 (14)	2 (25)	4 (17)
Foot	1 (13)	1 (14)	1 (13)	3 (13)
Hip	2 (25)	0 (0)	0 (0)	2 (9)
Knee	0 (0)	1 (14)	0 (0)	1 (4)

Percentages may not sum to totals because of rounding.

**Table 2:** Treatment-specific retention rates.

Timepoints	In-person + app n (%)	Virtual + app n (%)	Waitlist Control n (%)	Overall n (%)

Baseline	8 (100)	7 (100)	8 (100)	23 (100)
One month after 4-weeks APA) 1 M follow-up)	7 (88)	7 (100)	8 (100)	22 (96)
2 M follow-up	7 (88)	7 (100)	–	14 (93)^┼^
3 M follow-up	6 (75)	7 (100)	–	13 (87)^┼^

M = month

┼ denominator is the number of subjects randomized to the two APA groups.

**Table 3:** Treatment-specific adherence rate to study protocol.

	In-person + app Mean (SD) (Range) (% adherence)	Virtual + app Mean (SD) (Range) (% adherence)

Frequency using APA (# of times)		
Over the four-week intervention period	3.0 (0.33)	4.0 (1.65)
One month after 4-weeks APA (1 M follow-up)	1.7 (1.38) (0–3) (38)	3.1 (2.12) (0–6) (71)
2 M follow-up	1.6 (1.51) (0–3) (38)	3.0 (1.91) (0–5) (71)
3 M follow-up	1.8 (1.10) (0–3) (13)	3.3 (1.25) (1–5) (86)
Overall	2.5 (1.08)	3.6 (1.71)
Duration using APA per time (minutes)		
Over the four-week intervention period	3.1 (0.42)	3.7 (1.92)
One month after 4-weeks APA (1 M follow-up)	2.2 (1.58) (0.0–4.0) (50)	2.6 (1.27) (0.0–4.0) (71)
2 M follow-up	1.7 (1.60) (0.0–3.0) (50)	2.6 (1.27) (0.0–4.0) (71)
3 M follow-up	2.3 (1.48) (0.0–4.0) (25)	2.9 (0.38) (2.0–3.0) (86)
Overall	2.7 (1.11)	3.2 (1.64)
Duration using APA per day (minutes)		
Over the four-week intervention period	9.3 (1.60)	13.6 (7.07)
One month after 4-weeks APA (1 M follow-up)	5.4 (4.57) (0.0–12.0) (25)	9.6 (6.95) (0.0–18.0) (57)
2 M follow-up	4.7 (4.54) (0.0–9.0) (38)	9.1 (6.41) (0.0–16.0) (57)
3 M follow-up	5.1 (3.25) (0.0–8.0) (0)	9.4 (4.04) (3.0–15.0) (71)
Overall	7.6 (3.51)	11.7 (6.74)

M = month; Adherence = frequency using APA (# of times) at least 3 times, duration using APA per time (minutes) at least 3 min, and duration using APA per day (minutes) at least 9 min.

**Table 4:** Proportion of participants who continued to use APA on their own after the 4-week intervention.

	In-person + app n (%)	Virtual + app n (%)	Overall n (%)

Immediate post intervention			
Yes	4 (50)	7 (100)	11 (73)
No	3 (38)	0 (0)	3 (20)
Missing	1 (12)	0 (0)	1 (7)
One month after 4-weeks APA (1 M follow-up)			
Yes	4 (50)	5 (71)	9 (60)
No	3 (38)	2 (29)	5 (33)
Missing	1 (12)	0 (0)	1 (7)
2 M follow-up			
Yes	4 (50)	6 (86)	10 (67)
No	3 (38)	1 (14)	4 (26)
Missing	1 (12)	0 (0)	1 (7)
3 M follow-up			
Yes	5 (64)	6 (86)	11 (73)
No	1 (12)	1 (14)	2 (14)
Missing	2 (24)	0 (0)	2 (13)

M = month

**Table 5:** Proportion of measures that were completed.

Timepoints	In-person mAPA n (%)	Self-guided mAPA n (%)	Control n (%)	Overall n (%)

Baseline	8 (100)	7 (100)	8 (100)	23 (100)
One week after APA	7 (88)	6 (86)	8 (100)	21 (91)
Two weeks after APA^┼^	7 (88)	6 (86)	8 (100)	21 (91)
Three weeks after APA^┼^	7 (88)	7 (100)	8 (100)	22 (96)
Four weeks after APA^┼^	6 (75)	7 (100)	7 (88)	20 (87)
1 M follow-up	7 (88)	7 (100)	7 (88)	21 (91)
2 M follow-up	7 (88)	6 (86)	–	13 (87)*
3 M follow-up	6 (75)	7 (100)	–	13 (87)*

┼ only pain intensity was assessed; M = month

* the denominator is the number of subjects randomized to the two APA groups.

**Table 6:** Treatment difficulty with APA where 1 = no difficulty and 5 = extremely difficult.

Treatment Difficulty	In-person + app Mean (SD) (Range)	Virtual + app Mean (SD) (Range)

Frequency using APA		
One month after 4-weeks APA (1 M follow-up)	1.7 (0.76) (1–3)	2.1 (1.68) (1–5)
2 M follow-up	1.6 (0.79) (1–3)	2.3 (1.60) (1–5)
3 M follow-up	1.8 (1.30) (1–4)	1.9 (0.90) (1–3)
Duration using APA		
One month after 4-weeks APA (1 M follow-up)	2.0 (0.82) (1–3)	1.6 (0.79) (1–3)
2 M follow-up	1.6 (0.79) (1–3)	2.1 (1.46) (1–5)
3 M follow-up	1.8 (1.30) (1–4)	1.4 (0.53) (1–2)

M = month.

**Table 7:** Satisfaction with APA.

	In-person + app n (%)	Virtual + app n (%)	Overall n (%)

One month after 4-weeks APA (1 M follow-up)			
Completely satisfied	1 (13)	3 (43)	4 (27)
Somewhat satisfied	6 (75)	3 (43)	9 (60)
Not satisfied	0 (0)	1 (14)	1 (7)
Missing	1 (13)	0 (0)	1 (7)
2 M follow-up			
Completely satisfied	1 (13)	3 (43)	4 (27)
Somewhat satisfied	6 (75)	4 (57)	10 (67)
Not satisfied	0 (0)	0 (0)	0 (0)
Missing	1 (13)	0 (0)	1 (7)
3 M follow-up			
Completely satisfied	1 (13)	4 (57)	5 (33)
Somewhat satisfied	4 (50)	3 (43)	7 (47)
Not satisfied	0 (0)	0 (0)	0 (0)
Missing	3 (37)	0 (0)	3 (20)

Percentages may not sum to totals because of rounding; M = month.

**Table 8:** Proportion of participants who would recommend APA to family.

	In-person + app n (%)	Virtual + app n (%)	Overall n (%)

One month after 4-weeks APA			
1 M follow-up			
Yes	7 (88)	7 (100)	14 (93)
No	0 (0)	0 (0)	0 (0)
Missing	1 (12)	0 (0)	1 (7)
2 M follow-up			
Yes	7 (88)	7 (100)	14 (93)
No	0 (0)	0 (0)	0 (0)
Missing	1 (12)	0 (0)	1 (7)
3 M follow-up			
Yes	5 (63)	7 (100)	12 (80)
No	0 (0)	0 (0)	0 (0)
Missing	3 (37)	0 (0)	3 (20)

M = month.

## Data Availability

Data are available upon request to the corresponding author.
